# Single Chip-Based Nano-Optomechanical Accelerometer Based on Subwavelength Grating Pair and Rotated Serpentine Springs

**DOI:** 10.3390/s18072036

**Published:** 2018-06-26

**Authors:** Qianbo Lu, Jian Bai, Kaiwei Wang, Peiwen Chen, Weidong Fang, Chen Wang

**Affiliations:** State Key Laboratory of Modern Optical Instrumentation, Zhejiang University, Hangzhou 310027, China; lqb@zju.edu.cn (Q.L.); bai@zju.edu.cn (J.B.); cpwchen@126.com (P.C.); 21760105@zju.edu.cn (W.F.); mr_morning@163.com (C.W.)

**Keywords:** subwavelength grating pair, rotated serpentine, optomechanical, genetic algorithm, optimization, MOEMS

## Abstract

Optical coupling between subwavelength grating pairs allows for the precise measurement of lateral or vertical displacement of grating elements and gives rise to different types of displacement and inertial sensors. In this paper, we demonstrate a design for a nano-optomechanical accelerometer based on a subwavelength grating pair that can be easily fabricated by a single Silicon-on-insulator (SOI) chip. The parameters of the subwavelength grating pair-based optical readout, including period, duty cycle, thickness of grating and metal film, and the distance of the air gap, were optimized by combining a genetic algorithm and rigorous coupled wavelength analysis (RCWA) to obtain the optimal sensitivity to the displacement of suspended grating element and the acceleration. A corresponding mechanical design was also completed to meet the highly sensitive acceleration measurement requirement while considering the mechanical cross-axis sensitivity, dynamic range, bandwidth, and fabrication feasibility. This device was verified by both RCWA and finite-different-time-domain methods, and a tolerance analysis was also completed to confirm that it is able to achieve the extremely high optical displacement sensitivity of 1.8%/nm, acceleration-displacement sensitivity of 1.56 nm/m*g*, and acceleration measurement sensitivity of more than 2.5%/m*g*, which is almost one order of magnitude higher than any reported counterparts. This work enables a single SOI-based high performance accelerometer, and provides a theoretical basis and fabrication guides for the design.

## 1. Introduction

A considerable demand for high-performance inertial sensors has been growing in areas such as inertial navigation, seismic monitoring, and attitude control. Compared with traditional accelerometers that are based on capacitive, piezoresistive, and piezoelectric readouts, optical methods are promising due to their high sensitivity, immunity to electromagnetic interference, and remote sensing ability [1,2,3,4]. A large number of schemes using different optical techniques have been introduced, including interferometry [5,6], Fabry–Perot cavity [7,8], and evanescent wave (EW) coupling [9,10]. Among these methods, near-field evanescent wave coupling designs using subwavelength gratings have been verified to have the potential to increase the sensitivity beyond the other types [11,12,13,14].

However, current subwavelength gratings-based devices suffer from deficiencies and limitations. For example, the first reported EW coupling accelerometer [14] is difficult to fabricate, and controlling the relative position of two movable subwavelength gratings is difficult, since the response is sensitive to the dimensional parameters in addition to the in-plane motion. Yao [15] and Roger [10] used two subwavelength gratings to construct an equivalent grating with a larger period so that the diffraction could be observed, but the relative optical sensitivities of their designs were both less than 0.5%/nm, which means this scheme loses the benefits gained by using the EW coupling scheme. The optical displacement sensitivity is expressed by the unit “%/nm”, which means the percentage change of output light intensity when there is a relative displacement of one nanometer. Similarly, the unit “%/m*g*” means the percentage change of output light intensity when there is an acceleration of one millesimal gravitational unit. The highest reported lateral optical sensitivity was around 1.5%/nm based on a modified Keeler’s structure [11,16], and it also suffers from similar problems and has never been carried out due to the complicated fabrication process. 

A well-designed subwavelength grating can serve as a ultrabroadband mirror [17] and polarization reflector [18], and has been successfully applied in a tunable laser [19]. Inspired by the polarization selectivity of the subwavelength grating, we propose a nano-optomechanical accelerometer based on a newly designed subwavelength grating pair. The subwavelength grating pair was composed of an etched silicon grating covered with silver (Ag) film and the upper surface of the silicon substrate was covered with a complementarily periodic Ag film. This complementarily periodic Ag film effectively reduced the structural complexity and was able to address the fabrication problem. Furthermore, we recorded a profound sensitivity improvement. The subwavelength grating pair, which served as a highly sensitive optical displacement readout, was located at the center of a relatively bulky plate, which was also made by the device layer of a SOI wafer. Four rotated serpentine beams connected the plate to the frame, thereby suspending the plate and the silicon grating. Parameters of the subwavelength grating pair, such as the period, thickness, and duty cycle of the grating; spacing between the grating and bottom film; and the thickness of film were optimized along with the wavelength of the laser source to obtain the highest optical sensitivity. The settings of the elastic spring-mass structure, including the size of the plate and the dimensions and shape of the beams were optimized to obtain low mechanical cross-axis sensitivity and relatively high acceleration-displacement sensitivity by using analytical calculations and finite element modeling (FEM). Both the subwavelength grating pair and elastic structure can be fabricated with a SOI wafer. The thickness of the silicon grating and the gap between two subwavelength gratings can be tailored by the thickness of the device layer and the buried oxide layer, respectively. The tolerance of the subwavelength grating pair parameters and the elastic structure design are discussed in conjunction with the feasibility of the fabrication process.

The optimized subwavelength grating pair-based optical readout can possibly attain the optical displacement sensitivity of approximately 2%/nm, which outperforms the previously reported counterparts. Combined with the corresponding mechanical design that has acceleration-displacement sensitivity of 1.56 nm/*g*, this sensor can realize high acceleration sensitivity of 2.8%/m*g*, proving itself as a potential high-performance and easily fabricated inertial sensor. 

## 2. Subwavelength Grating Pair Design

**Figure 1:**
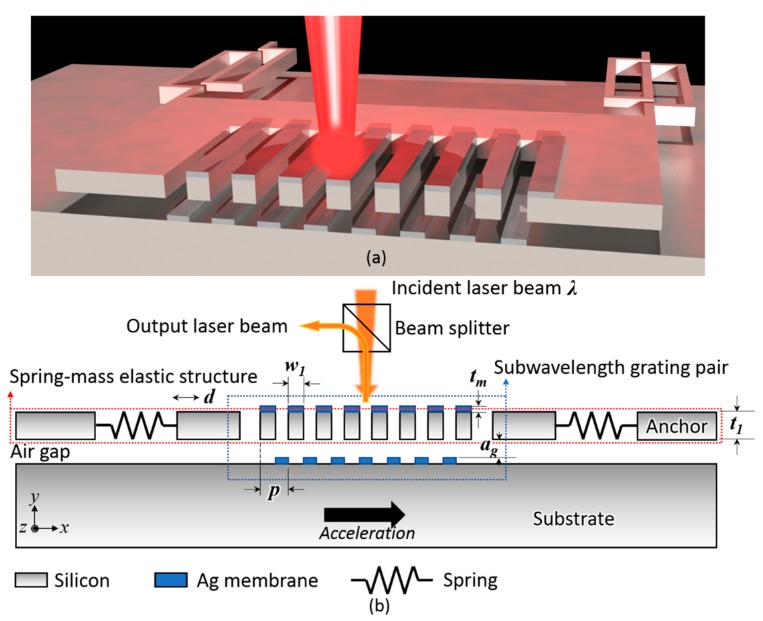
(**a**) Schematic diagram of the subwavelength grating pair-based optomechanical accelerometer along with; (**b**) its cross section view.

Figure 1 displays the schematic diagram of our subwavelength grating pair-based optomechanical accelerometer that contains a subwavelength grating pair-based optical displacement readout and a spring-mass micromachined structure. The optical readout consists of a laser source, a beam splitter, and a subwavelength grating pair, and the subwavelength grating pair was composed of an etched silicon grating coated with Ag film and the upper surface of the silicon substrate covered with periodic Ag film grating. These two Ag films were periodically complementary, which could be evaporated in a process. These two subwavelength gratings were separated by an air gap, whose thickness could be tailored by the thickness of the sacrificial layer of a SOI wafer. The upper subwavelength grating was suspended by some designated springs and was movable along the *x*-axis. When a Transverse Electric (TE) mode laser illuminated the subwavelength grating pair normally, the intensity of the zeroth-order reflected light would change considerably with the motion of the upper grating. Combining this with feasible acceleration-displacement sensitivity, this device makes it as a potential candidate for detecting small acceleration with high sensitivity.

**Figure 2:**
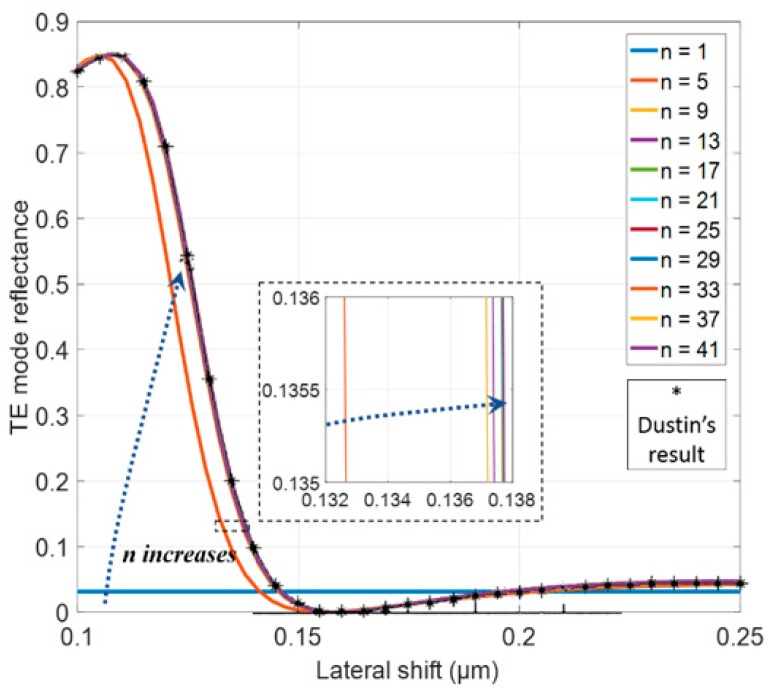
TE mode reflectance versus lateral shift of movable grating versus an increasing number of harmonics. The solid lines represent our simulation results and the asterisks represent Dustin’s results.

We used rigorous coupled wavelength analysis (RCWA) to simulate the electrical field distribution and reflected intensity. In order to verify the correctness of this method, we reproduced Dustin’s simulation by using the same setting parameters and compared our result to Dustin’s result that was verified by their experiment [12]. The simulation accuracy is mainly affected by the number of harmonics used in the calculation. Herein, we used 41 harmonics, including 20 positive, 20 negative, and one zeroth-order. The comparison of our simulation results with different numbers of harmonics and Dustin’s result is shown in Figure 2, which indicates that 41 harmonics were sufficient to attain an accurate result. 

For our design, the input laser was set to a TE polarization planar wave. The parameters of the subwavelength grating pair-based optical readout were: the period of upper silicon grating (*p*), duty cycle of the silicon grating (*dc* = *w*_1_/*p*), thickness of the silicon grating (*t*_1_), thickness of the film (*t_m_*), air gap between the silicon grating and the upper surface of the bottom film (*a_g_*), and wavelength of the incident laser (*λ*). The material of the film could be adjusted by changing the refractive index, and we chose Ag due to its common fabrication process and low refractive index. A genetic algorithm was used combined with RCWA to optimize these parameters, where the objective function was defined as the slope of the curve of reflectance versus the lateral displacement. This algorithm helped to avoid running into a local optimization solution and dramatically reduced the computation time compared with multiparameter sweep.

Considering the feasibility and dimensions of the SOI wafer, we set the initial upper and lower limit of these parameters as listed in Table 1. The parameters for the genetic algorithm were set as follows: Pareto fraction was 0.3, size of the population was 200, generation number was 200, and function tolerance was 10^−100^. After roughly positioning the optimal sensitivity, we narrowed the range of the parameters, then used an iterative algorithm to search for the optimal solution. The refractive index of the material was changed with the wavelength.

**Figure 3:**
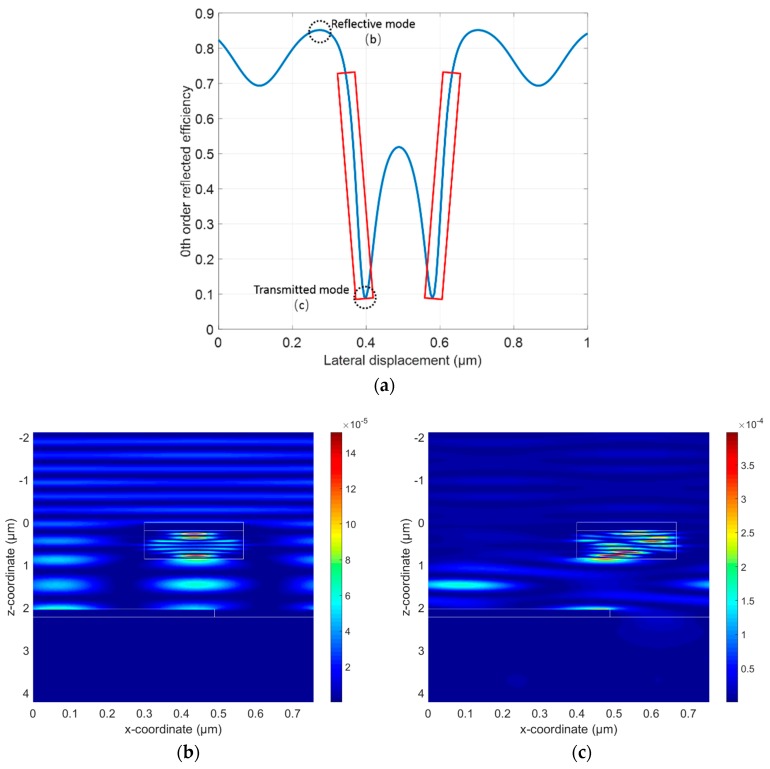
(**a**) Curve of the zeroth order reflectance as a function of displacement of the silicon grating along the *x*-axis for the optimal optical design; (**b**) reflective mode when the reflectance was the maximum in one period; the lateral shift of the upper grating was around 300 nm; (**c**) transmitted mode when the reflectance was the minimum in one period, the lateral shift of the upper grating was around 390 nm.

The optimal values of the parameters are outlined in Table 1. The linewidth of the silicon grating and the upper Ag film *w_1_* was calculated as 267.6 nm. Hence the grating pair was a subwavelength grating pair. The relationship between the zeroth order reflectance and the lateral displacement along the *x*-axis is depicted in Figure 3a. The optical displacement sensitivity is defined as *s_o_* = Δ*R*/Δ*d*, in which *R* is the zeroth reflectance and *d* is the lateral displacement along the *x*-axis. In the marked red square region, the optical sensitivity was as high as 1.8%/nm, which is the highest ever reported level. Figure 3b,c show the electric field intensity distribution of the reflective mode and transmitted mode, respectively. A visualization movie is included in the Supplementary Materials, which illustrates the change in the electric field along with the lateral movement of the silicon grating along the *x*-axis.

**Figure 4:**
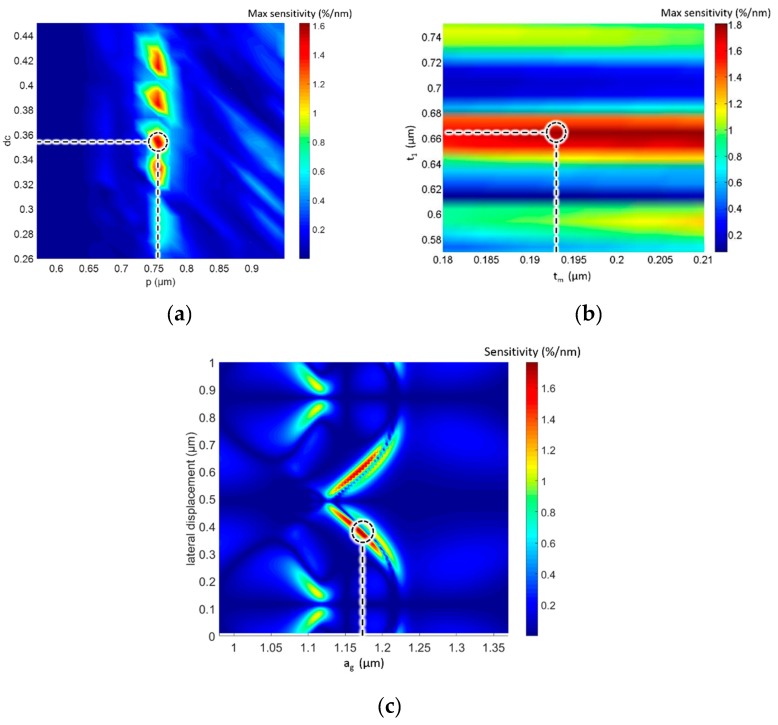
Contour map of the relationship between (**a**) the maximum optical sensitivity and period and duty cycle; (**b**) the maximum optical sensitivity and thickness of silicon grating and the film; and (**c**) the optical sensitivity and lateral displacement and air gap.

A multiparameter sweep was also performed in small intervals to determine whether the optimized parameters were the optimal ones. However, sweeping all six variables was impractical because 100^6^ times of RCWA calculation would be required if we set 100 steps for each parameter, which would take millions of years. Hence, we changed the parameters in pairs and plotted the contour of the maximal optical sensitivity in terms of two variables. Figure 4 indicates that this optimized solution did have peak sensitivity in the swept regions. In addition, different parameters had different impacts on the sensitivity. Period (*p*), duty cycle (*dc*), air gap (*a_g_*), and thickness of the silicon and grating (*t*_1_) had considerable impacts on the maximal sensitivity, whereas the variations in the thickness of film (*t_m_*) had relatively less impacts on the sensitivity. This provides understanding about the weight of influence of all the parameters and establishes the tolerance analysis that can guide the fabrication process.

**Figure 5:**
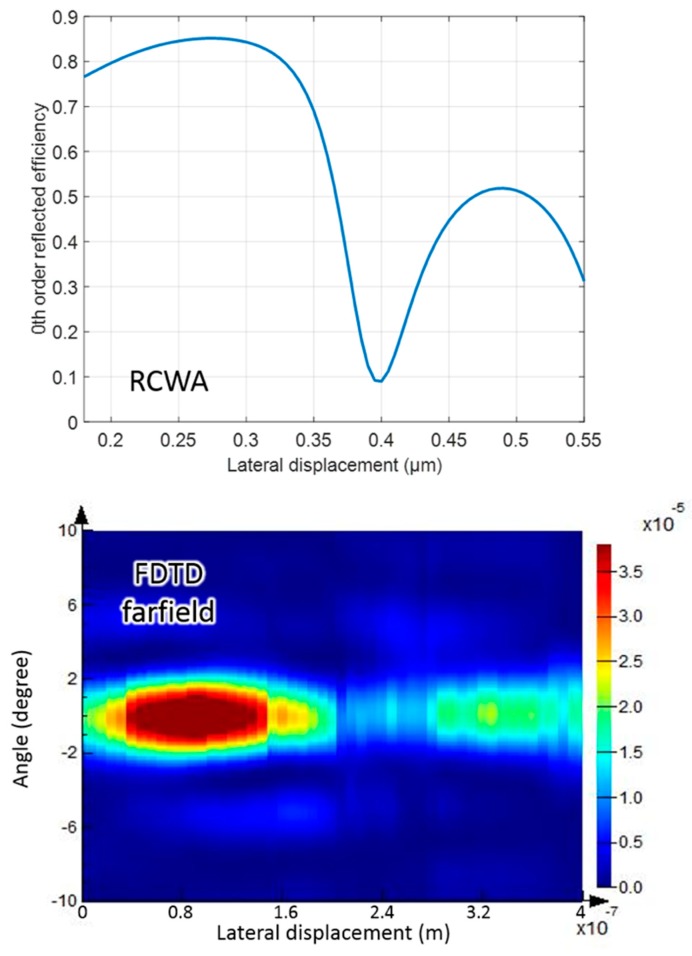
Comparison of the rigorous coupled wavelength analysis (RCWA) simulation result with the finite-different-time-domain (FDTD) simulation result for the identical optimal settings. The far field electric intensity of the FDTD result was recorded from +10° to −10°.

The finite-different-time-domain (FDTD) method was also implemented for comparison with the results obtained by RCWA simulation. All the parameters were identical to the optimal values listed in Table 1 and a normal incident TE mode planar wave was chosen as the laser source. The minimum mesh step of FDTD simulation was 10^−14^ m. We simulated the far field electric intensity distribution, where the electric field in range of positive +10° and −10° is presented in Figure 5, which denotes the zeroth order reflectance. The distribution was consistent with the RCWA result, confirming the validity of our simulation and optimized solution.

**Figure 6:**
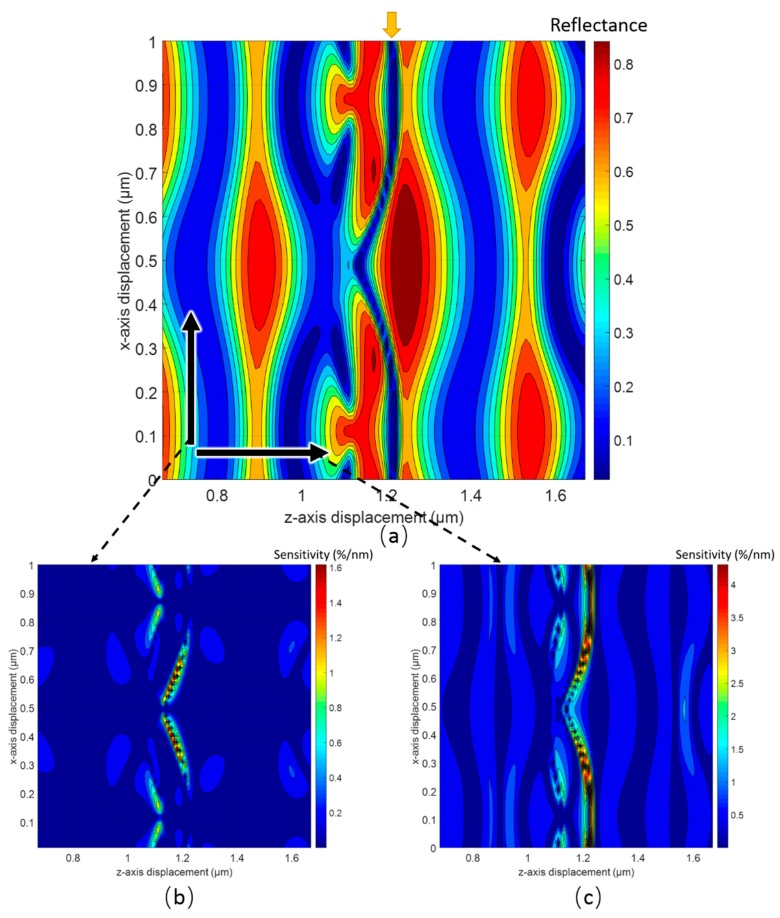
(**a**) Contour plot of the reflectance as a function of both in-plane and out-of-plane displacement; (**b**) contour plot of the optical sensitivity along the *x*-axis as a function of both in-plane and out-of-plane displacement; (**c**) contour plot of the optical sensitivity along the *z*-axis as a function of both in-plane and out-of-plane displacement.

The sensitivity of the out-of-plane motion for our design is worth noting. Figure 6a shows a contour map of the zeroth reflectance versus the *x*-axis and *z*-axis displacement of the silicon grating. The split, marked by a red arrow, breaks the periodicity and leads to the peak optical sensitivity. Not only is the sensitivity along the *x*-axis very high, but the sensitivity along the *z*-axis is also extremely high (~5%/nm). As such, this type of optical readout is also a promising candidate as an out-of-plane sensor.

## 3. Mechanical Design

### 3.1. Rotated Serpentine Springs

**Figure 7:**
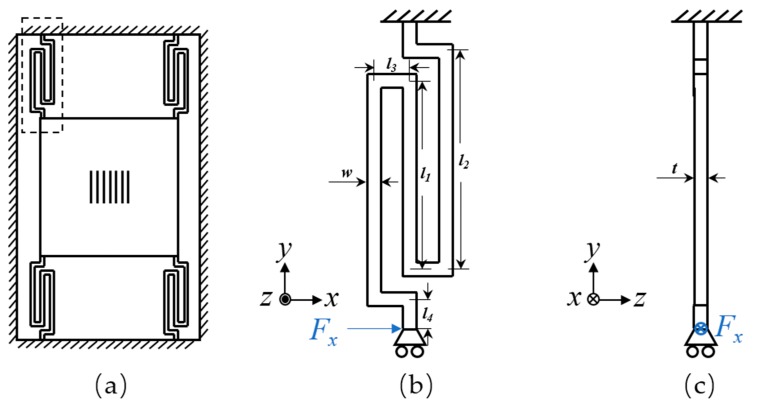
(**a**) Schematic of the elastic micromachined structure with four rotated serpentine springs; (**b**) top view and (**c**) cross-sectional view of a rotated serpentine spring with a guided end.

The optimal mechanical design, with a rotated serpentine spring-mass structure, was used to achieve reasonable mechanical sensitivity and dynamic range as well as low mechanical cross-axis sensitivity combined with the optimal optical readout. As illustrated in Figure 7a, a bulky silicon plate with grating pattern, which serves as a proof mass, was connected to the silicon frame by four symmetrically distributed rotated serpentine springs. The grating was nestled in the center of the bulky plate, whereas the bulky plate was suspended by releasing the sacrificial layer. The thickness of the plate, springs, and the silicon frame were equal to the thickness of the single-crystal silicon layer (*t*_1_). 

The rotated serpentine spring was chosen due to its low spring constant (high acceleration-displacement sensitivity), small occupation of area, and relatively low cross-axis sensitivity [20]. The schematic diagram of a rotated serpentine spring with a guided end is shown in Figure 7b,c. We simplified the case by taking advantage of the geometric symmetry of the structure during the calculation of the spring constant of each spring *k_x_*. Because the dimension of the proof mass was far larger than that of the springs, the overall elastic coefficient of the device could be approximated to 4*k_x_*. The end of the spring was bound to the frame without rotation and displacement. The rotated serpentine spring is a centrosymmetric structure; thus, the dimensional parameters can be simplified to *l*_1_, *l*_2_, *l*_3_, and *l*_4_, and width *w*, and thickness *t*. Herein, we define the *x*-axis as the sensitive axis of the device, so the spring constant along the *x*-axis can be calculated by integrating the strain energy density along the beam and using the unit-load method [21]:

(1) $$k_{x}=\frac{k_{\theta_{z}x}{}^{2}k}{k_{\theta_{z}x}{}^{2}-k_{\theta_{z}}k}，$$

in which

(2) $$\begin{array}{l} k=3EI_{z}/\left(\begin{array}{l} 3l_{3}\left(l_{4}{}^{2}+{\left(l_{4}+l_{2}-l_{1}\right)}^{2}+{\left(l_{4}+l_{2}\right)}^{2}+{\left(l_{4}+2l_{2}-l_{1}\right)}^{2}\right)\\ +2\left({\left(l_{4}+l_{2}\right)}^{3}-{\left(l_{4}+l_{2}-l_{1}\right)}^{3}\right)+{\left(2l_{4}+2l_{2}-l_{1}\right)}^{3} \end{array}\right),\\ k_{\theta_{z}}=M_{z}/\theta_{z}\\ =EI_{z}/\left(3l_{1}+2\left(l_{2}-l_{1}\right)+2l_{4}+4l_{3}\right),\\ k_{\theta_{z}x}=F_{x}/\theta_{z}\\ =2EI_{z}/\left(2l_{1}\left(2l_{4}+2l_{2}-l_{1}\right)+{\left(2l_{4}+2l_{2}-l_{1}\right)}^{2}+2l_{3}\left(4l_{4}+4l_{2}-2l_{1}\right)\right). \end{array}$$

*E* is the anisotropic Young’s modulus of single-crystal silicon in nanometer-scale (for Young’s modulus along the *x*-axis axis here, *E_x_* = 169 GPa [22,23]; details can be found in the Supplementary Materials), *M_z_* is the torsion moment around the *z*-axis, and *I_z_* = *tw*^3^/12 is the moment of inertia with respect to the *z*-axis. *k*, *k_θz_*, and *k_θzx_* are elements of 6 × 6 spring constant matrix (Supplementary Materials). To achieve high sensitivity along the *x*-axis and relatively low mechanical cross-axis sensitivity, *l*_1_ and *l*_2_ were set to be much larger than *l*_3_ and *l*_4_, whereas *l*_1_ and *l*_2_ were close in value.

**Figure 8:**
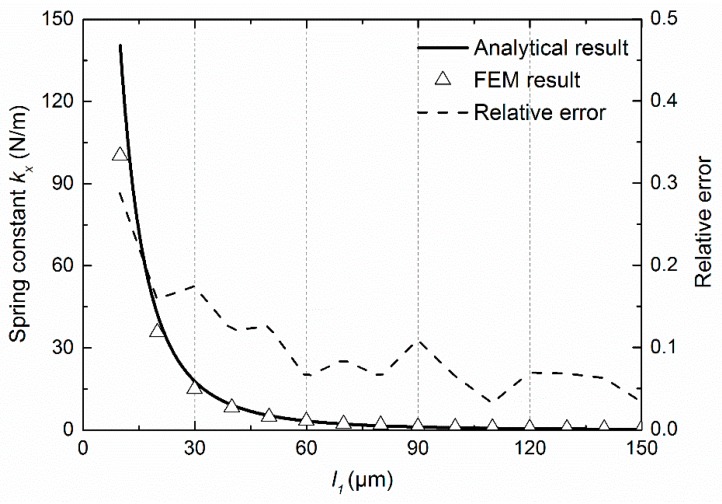
Curves of spring constant along the *x*-axis as a function of *l*_1_ by both analytical calculation and finite element modelling (FEM) simulation along with the relative error as a function of *l*_1_, which is defined by (*k_FEM_* − *k_anal_*)/*k_FEM_*. Solid lines represent the approximate analytical results, triangles represent FEM simulation results, and dashed lines represent the relative errors. Red, black, and blue denote *w* = *t* = 0.5, 1, and 2 μm, respectively.

Figure 8 compares the analytical result with the FEM result, in which the solid line represents the analytical result of spring constant versus the length *l_1_* calculated from Equation (1), the dashed line represents the relative error that are defined by (*k_FEM_* − *k_anal_*)/*k_FEM_*, whereas triangles represent the FEM simulation results of the spring constant obtained by using an anisotropic elastic matrix of stiffness form [24]. *l*_1_ is the variable and *l*_2_ − *l*_1_, *l*_3_, *l*_4_, *w,* and *t* are all fixed to 1 μm. The analytical calculation and FEM simulation were completed by setting *l*_2_ − *l*_1_, *l*_3_, and *l*_4_ to 3, 4, and 4 μm, respectively. The value calculated from the analytical expression coincided well with the FEM simulation when *l*_1_ was much larger than *l*_2_ − *l*_1_, *l*_3_, and *l*_4_. Typically, the relative error was smaller than 20% when *l*_1_ was 5 times more than *l*_2_ − *l*_1_, *l*_3_, and *l*_4_. The deviation shown in Figure 8 was due to the FE model containing the proof mass, with dimension of 5 mm × 5 mm × 1 μm, and the load was the global acceleration. Thus, the simulated spring constant was the elastic performance of the whole system, which would change with the increase in the dimension of the springs, since the weight and the inertial force cannot be neglected. Furthermore, the length of the beam was approximated as constituting the spring as the distance between the centers of two consecutive corners during analytical derivation. 

Similarly, the spring constant along two different axes was derived. We obtained the approximate analytical expressions as:

(3) $$k_{y}≃\frac{Ew^{3}t}{24l_{1}l_{3}{}^{2}},$$

(4) $$k_{z}≃\frac{Ewt^{3}}{3l_{1}{}^{3}}.$$

Equations (2)–(4) can be applied to predict the spring constant and thus the acceleration-displacement sensitivity and mechanical cross-axis sensitivity. Combined with the sensitivity of the aforementioned optical readout and fabrication feasibility, the optimal mechanical design could be obtained in terms of different target performances.

The acceleration sensitivity (*s_a_*, %/m*g*) is termed as the optical displacement sensitivity (*s_o_*, %/nm) multiplied by the acceleration-displacement sensitivity (*s_a-d_*, nm/*g*)

(5) $$s_{a}=s_{o}\cdot s_{a-d}.$$

The acceleration-displacement sensitivity is inversely proportional to the spring constant. The target was to achieve high sensitivity along the sensitive axis and relatively low cross-axis sensitivity, which involves *k_x_*/*k_i_*, where the subscript denotes the appropriate axis. Fortunately, the optical readout was insensitive to the displacement along the *y*-axis if the grating area was larger than the beam size, and the acceleration-displacement sensitivity along the *y*-axis was far smaller than that along the *x*-axis under the premise that *l*_3_ << *l*_1_. Hence, the cross-axis sensitivity of the *y*-axis could be neglected. For the cross-axis sensitivity of the *z*-axis, slender beam geometry (*w* < *t*) should be used because *k_x_*/*k_i_* = (*w*/*t*)^2^. The ratio can be adjusted in terms of the target performance and sensitive axis. Here we set *w* < *t* because we viewed the *x*-axis as the sensitive axis.

**Figure 9:**
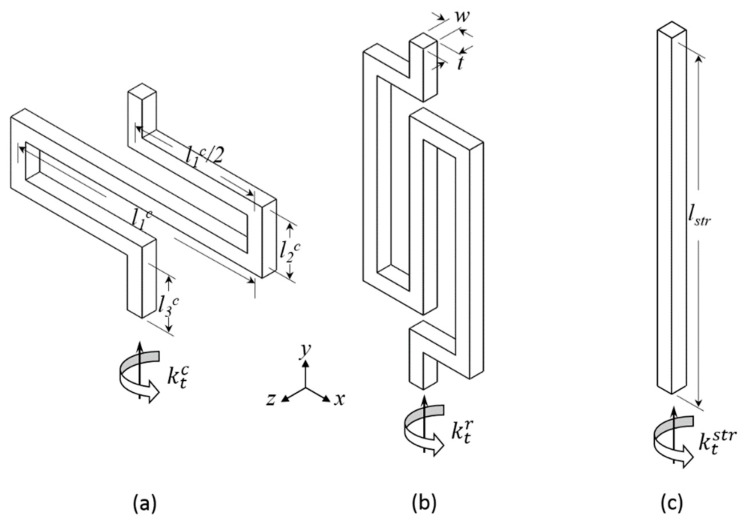
Schematics of (**a**) classical serpentine; (**b**) rotated serpentine; and (**c**) straight beam springs with dimensional parameters.

Torsion occurs along the axis of the spring. Our focus was the torsional stiffness around the *y*-axis because it was concerned with the rotation of the grating and would change the final optical output considerably [25]. Three specific springs were compared: straight beam, classical serpentine spring, and rotated serpentine spring, as illustrated in Figure 9. Equations (6)–(10) provide the expressions of the spring constant and torsion constant of each spring based on energy method, where $k_{x}^{c}$, and $k_{x}^{str}$ are the spring constants along the *x*-axis of classical serpentine spring and straight beam, respectively; $k_{t}^{r}$, $k_{t}^{c}$, and $k_{t}^{str}$ are the torsion constants of three springs around the *y*-axis; *G* denotes the shear modulus of the material; and $l_{1}^{c}$ and *l^str^* are the lengths marked in Figure 9. The derivation process can be found in the Supplementary Information.

To verify the torsion-resistance of the rotated serpentine spring, we provide a simple calculation: assuming all settings had the same beam width and thickness and let *w* = *t* = 0.1 mm. A set of specific dimension parameters are listed in Table 2, which were used to obtain an identical spring constant. Substituting these values into Equations (6)–(10), we found that the torsion constant of the rotated serpentine spring was much smaller than that of the classical serpentine spring, and even smaller than that of the straight beam, as shown in Table 2. This implies that the rotated serpentine spring can effectively suppress the rotation due to torque without compromising the acceleration-displacement sensitivity along the sensitive axis. As a result, we chose the rotated serpentine spring as the elastic suspension structure in our scheme design.

(6) $$k_{t}^{r}≃\frac{3Gw^{3}t}{64l_{1}},$$

(7) $$k_{x}^{c}≃\frac{Ew^{3}t}{152l_{2}^{c}{}^{2}l_{1}^{c}},$$

(8) $$k_{t}^{c}≃\frac{Ewt^{3}}{24l_{1}^{c}}，$$

(9) $$k_{x}^{str}≃\frac{4Ew^{3}t}{l_{str}{}^{3}},$$

(10) $$k_{t}^{str}≃\frac{9Gwt^{3}}{128l_{str}}.$$

Typical sensitivities of subwavelength grating pair-based schemes are 0.002%/m*g* as reported by Rogers [26] and 0.46%/m*g* as reported by Yao [15]. The former uses a much more rigid elastic structure (*k* ~ 1000 N/m) and the latter uses a relatively softer structure (*k* ~ 20 N/m), whereas their optical sensitivities are of the order of approximately 0.2%/nm. Combined with the substantially improved optical sensitivity of 1.8%/m*g*, we set our acceleration sensitivity target to 2%/m*g*, corresponding to the acceleration-displacement sensitivity of 1.11 nm/mg. This acceleration-displacement sensitivity could be realized by a feasible mechanical design. The main parameters included *l*_1_, *w*, *t*, and the dimensional parameters of the bulky plate, but they were limited by the bandwidth, dynamic range, and fabrication feasibility. Since *t* is determined by the grating thickness in the optical design, the remaining variables were *l*_1_, *w*, and the dimensional parameters of the bulky plate.

### 3.2. Limitation from Fabrication Feasibility

**Figure 10:**
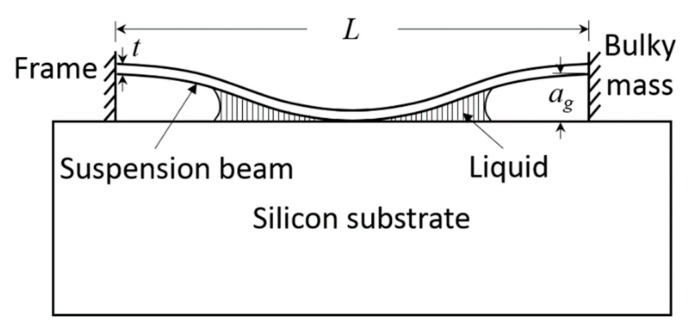
Cross-section view of a clamped–clamped beam adhering to the substrate.

Mechanical stability and adhesion in the surface micromechanical structure are critical constraints for the dimensions of soft suspensions [27], which are made by the thin single-crystal layer. We focused on the release-related adhesion during wet etching release and in-use adhesion of spring beams because they are the softest components in the device, and are therefore the key to fabrication feasibility. The release-related anti-stiction condition is given by energy method according to Mastrangelo [28]:

(11) $$\frac{128Eg_{a}{}^{2}t^{3}}{15\gamma_{l}\cos\theta_{c}L^{4}\left(1+t/w\right)}\left[1+\frac{2\sigma_{R}L^{2}}{7Et^{2}}+\frac{108}{245}{\left(\frac{g_{a}}{t}\right)}^{2}\right]>1,$$

where $\gamma_{l}$ is the liquid surface tension, $\theta_{c}$ is the contact angle of the liquid on the solid surface, $\sigma_{R}$ is the residual stress of the beam, *a_g_* is the air gap which equals the thickness of the sacrificial layer, and *L* is the final equivalent length of the beam, as shown in Figure 10. This model simplifies the rotated serpentine spring beam to a straight beam. The most conservative estimation is that the whole length of the rotated serpentine spring contributes to the stiction in this model, which means the maximal equivalent length of the suspension beam *L_max_* is approximate to 3*l*_1_ because *l*_1_ is far larger than *l*_3_ and *l*_4_, and is close to *l*_2_. We define $a_{1}=\frac{128Eg_{a}{}^{2}t^{3}}{15\gamma_{l}\cos\theta_{c}\left(1+t/w\right)};b_{1}=\frac{2\sigma_{R}}{7Et^{2}};c_{1}=1+\frac{108}{245}{\left(\frac{g_{a}}{t}\right)}^{2}$. Then, the maximal equivalent length of the suspension beam can be obtained:

(12) $$L_{\max}=\frac{a_{1}b_{1}+\sqrt{a_{1}{}^{2}b_{1}{}^{2}+4a_{1}c_{1}}}{2}.$$

**Figure 11:**
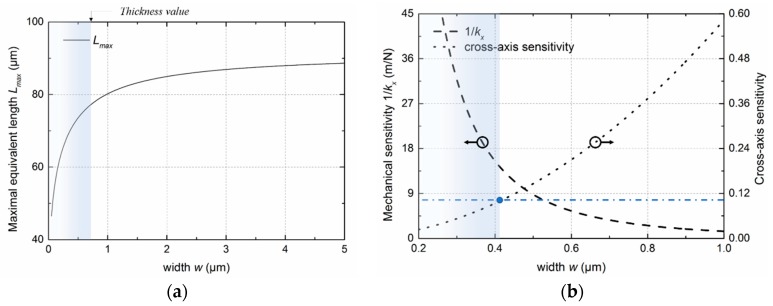
(**a**) Maximal equivalent length of anti-stiction condition as a function of the width; (**b**) mechanical sensitivity and cross-axis sensitivity derived from the maximal equivalent length as a function of the width.

Likewise, the in-use anti-stiction condition is expressed as:

(13) $$\frac{128Eg_{a}{}^{2}t^{3}}{5\gamma_{s}L^{4}}\left[1+\frac{4\sigma_{R}L^{2}}{21Et^{2}}+\frac{256}{2205}{\left(\frac{g_{a}}{t}\right)}^{2}\right]>1,$$

where $\gamma_{s}$ is the interfacial adhesion energy of per unit contact area. Equation (11) is a stronger restriction if $\gamma_{s}$ and $\gamma_{l}$ have the same order [29]. Herein, we considered the worst case scenario where $\theta_{c}$ = 0, $\sigma_{R}$ = 0 MPa, and $\gamma_{l}$ = 22 mJ/m^2^ which is close to isopropyl alcohol’s tension, which can be further decreased by using liquid CO_2_ or even supercritical CO_2_. *t* and *a_g_* were fixed to 0.7 μm and 1.2 μm in terms of the optimal optical design, respectively. Figure 11a depicts the maximal equivalent length *L_max_* = 3*l*_1_ as a function of variable *w*. The boundary value marked in Figure 11 denotes the thickness of the beam, which should be smaller than the width to reduce the cross-axis sensitivity. Combining this calculated *l*_1_ and Equations (1) and (2) and the definition of mechanical cross-axis sensitivity, we can obtain the curves of mechanical sensitivity (denoted by 1/*k_x_*) and cross-axis sensitivity versus width *w*, as shown in Figure 11b. To achieve relatively low cross-axis sensitivity (e.g., less than 10%), *w* should be smaller than 0.5 μm. The suitable region of the width is marked in Figure 11b. After determining the width, *l*_1_ and the corresponding spring constant along the sensitive axis was obtained.

In conjunction with the precision of lithography, 0.4 μm was chosen as the width of the rotated serpentine spring, and the corresponding *l*_1_ was 25 μm. The overall spring constant of the four rotated serpentine springs-mass system was calculated as around *k_o_* = 0.3 N/m (4*k_x_*). The dimension of the bulky plate was determined using the target acceleration-displacement sensitivity along with the dynamic range and bandwidth that involve maximal stress and resonant frequency, respectively. Because the target was approximately 1 nm/m*g*, the mass of the bulky plate should be larger than 3 × 10^−8^ kg.

(14) $$m_{c}=k_{o}\times \frac{d}{a}=k_{o}\times s_{a-d}.$$

**Figure 12:**
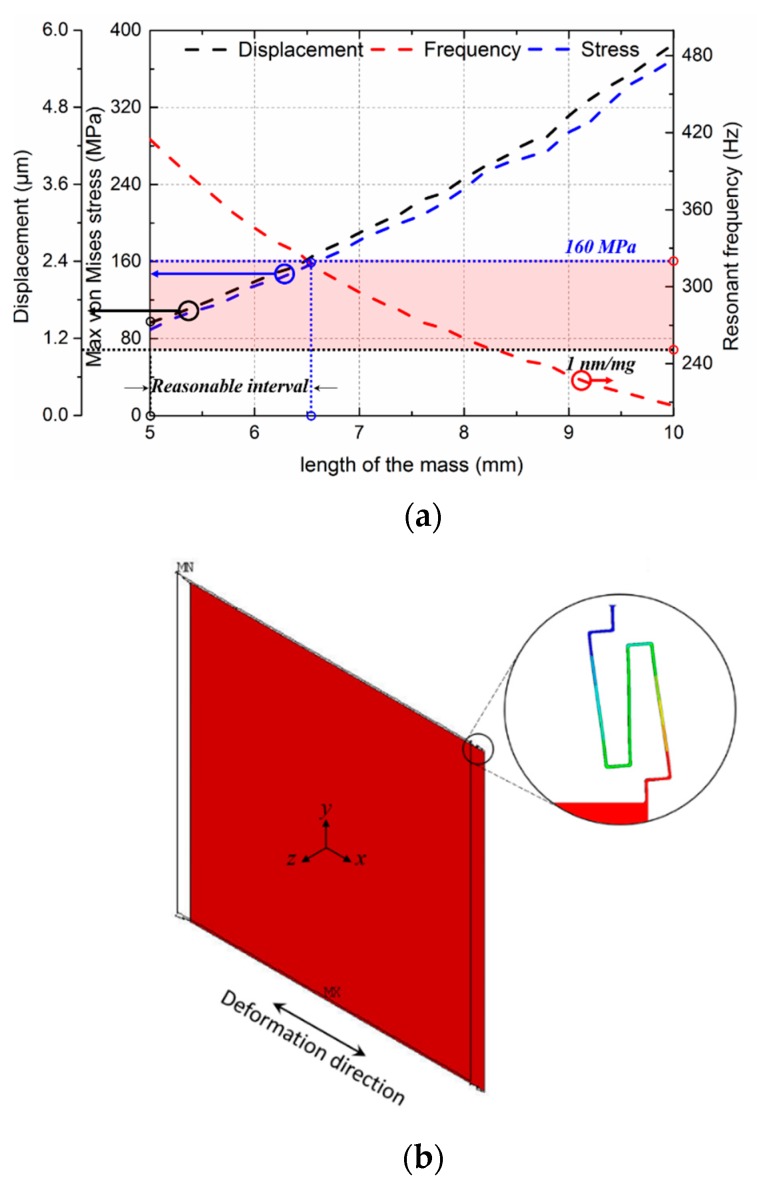
(**a**) Relationship between displacement along the *x*-axis, the maximum von Mises stress, the resonant frequency, and the length of the bulky plate when the structure was subjected to one *g* acceleration along the *x*-axis, where dotted lines mark the reasonable interval of the length according to the target performance and tensile strength of the material; (**b**) second modal deformation of the structure. Inset: deformation of a suspension beam in this mode.

Assuming the bulky plate was a square plate, we therefore set the length of the plate to be larger than 4.3 mm. The acceleration-displacement sensitivity, maximal stress, and the resonant frequency as functions of the length of the bulky plate were evaluated using the FEM method with an inertial load of one unit of gravity (1 *g*) along the *x*-axis, as shown in Figure 12a. Chamfering with a radius equaling *w* was introduced to diminish the concentrated stress. The resonant frequency is the frequency of the second mode, whose deformation was along the *x*-axis, as shown in Figure 12b. The black, blue, and red dashed curves represent the displacement, maximal von Mises stress, and frequency versus length of the bulky plate. According to the target acceleration-displacement sensitivity and the fracture strength of silicon, which were 1 nm/m*g* and 160 MPa (around one-tenth of the limit of 1.69 GPa [30]), respectively (marked by dotted lines), the reasonable interval of the length could be obtained. The corresponding resonant frequency was in the range of 320 to 450 Hz and did not change much when the length was located in this interval. To provide some allowance, we set the length of bulky plate to 6 mm. Given the above, the dimensional parameters and mechanical performances of the mechanical structure were determined, as listed in Table 3. The maximum von Mises stress was around 150 MPa when it was subjected to an acceleration of 1.5 *g* along the sensitive axis; when the applied acceleration increased to 16 *g*, the maximum stress became 1.6 GPa. Hence, the maximum load (mechanical measurement range) was determined as 1.5 *g* in consideration of 10% fracture strength of silicon or 16 *g* in consideration of 100% fracture strength of silicon.

## 4. Overall Performance and Tolerance Discussion

**Figure 13:**
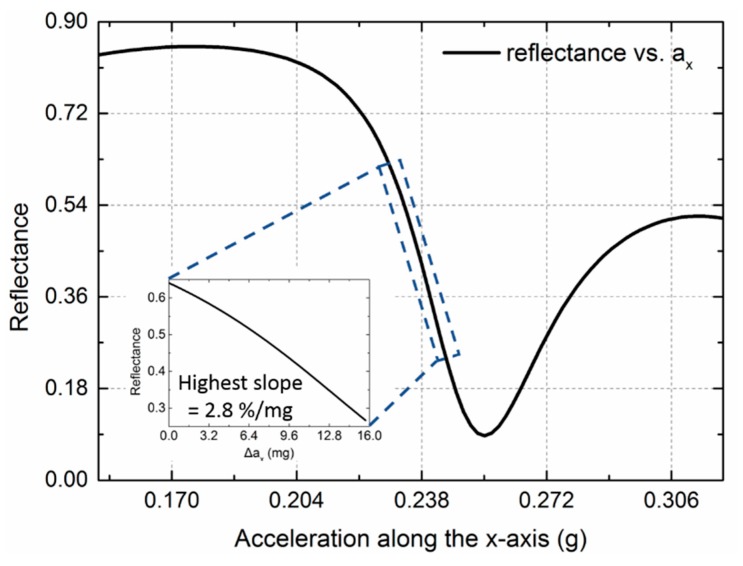
Curve of the zeroth reflectance as a function of the acceleration along the *x*-axis. Inset: enlarged view of the linear region, which indicates the highest slope can be as high as 2.8%/m*g*.

Combining the optimized optical readout and the mechanical design, we finally obtained the acceleration measurement sensitivity of the overall device. The response of the zeroth-order reflectance versus the applied acceleration is depicted in Figure 13. The highest slope was 2.8%/m*g*, which means our design has the potential to improve the current acceleration measurement sensitivity of subwavelength grating-based accelerometers by almost one order of magnitude compared with the typical sensitivities of 0.46%/m*g*. Although the linear region seems to be small, it is a trade-off between the sensitivity and the linear range for an optical technique-based accelerometer. Other optical accelerometers are able to achieve higher acceleration measurement sensitivity based on ultra-soft structure and interferometric method [31]; however, their linear range is even smaller than this scheme. The compared optical accelerometers, such as the subwavelength grating-based sensor presented by Sandia Laboratory [11,12] and diffraction grating-based design presented by Loh in MIT [32], feature a similar linear range (i.e., around 100 nm). Moreover, this single-chip design can easily introduce electrostatic servo combs by surface micromachining to address the feedback issue. 

**Figure 14:**
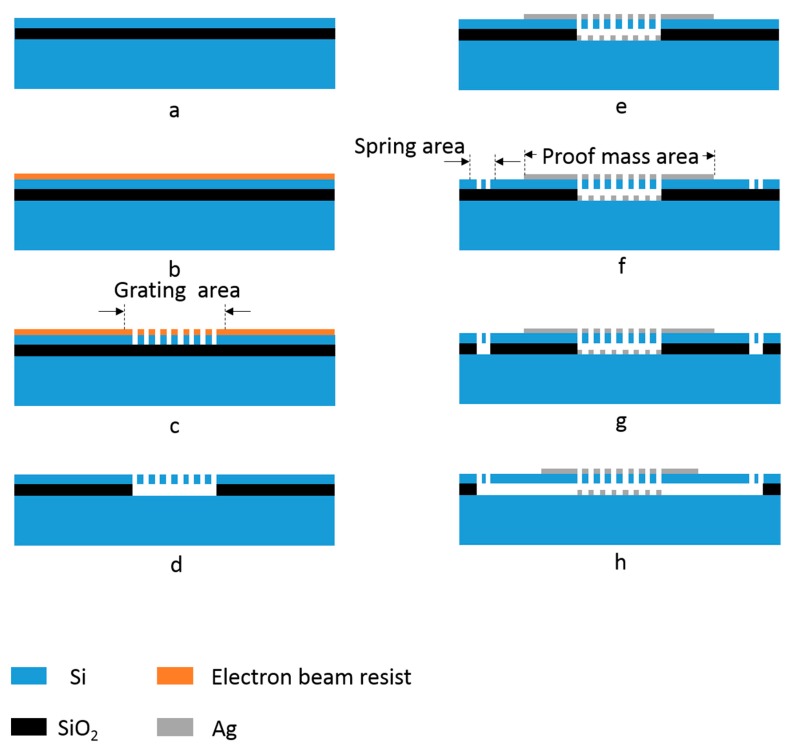
Process flow for fabrication of the micromachined structure. (**a**) Starting mirror polished SOI wafer; (**b**) spin coat the electron beam resist; (**c**) make the pattern of grating lines by electron beam lithography (EBL) and etch the device layer to the buried oxide layer by inductively coupled plasma (ICP) etching; (**d**) release the grating area by buffered oxide etch (BOE); (**e**) deposit Ag and then use lift-off to pattern the upper metal film; (**f**) also use EBL and ICP to pattern and etch the springs; (**g**) release the suspended springs by BOE; (**h**) release the proof mass to make the whole device suspended.

**Figure 15:**
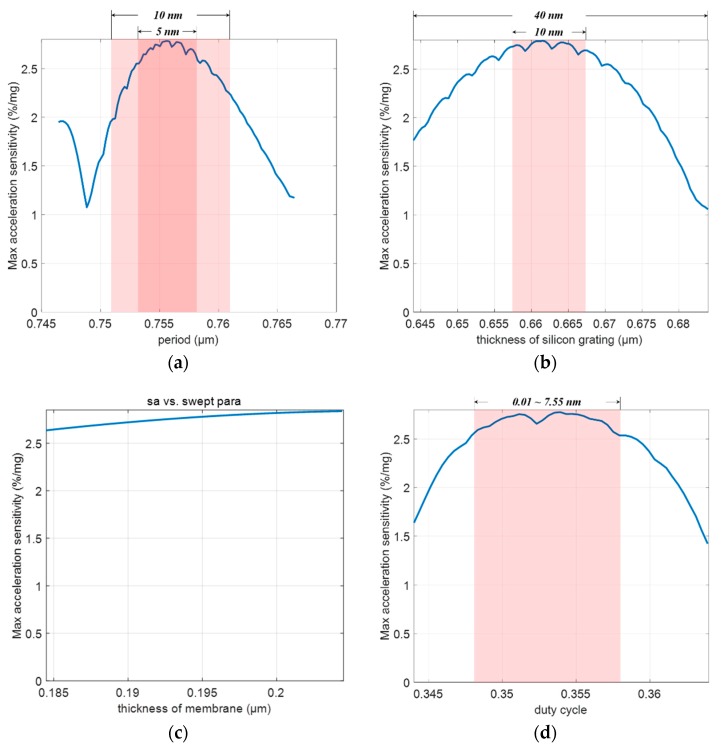
Maximum acceleration measurement sensitivity as a function of: (**a**) The period of silicon grating, with sensitivity being higher than 2%/m*g* with a tolerance of 10 nm; (**b**) the thickness of silicon grating, with sensitivity that can be higher than 2%/m*g* with a tolerance of 20 nm; (**c**) the film thickness, which has little impact on the sensitivity; and (**d**) the duty cycle, where the sensitivity can be higher than 2.5%/m*g* with a tolerance of 0.01.

Next, we completed a tolerance analysis to help understand the performance and guide device fabrication. A feasible fabrication process flow is provided in Figure 14. Figure 15a presents the maximal acceleration measurement sensitivity as a function of the period of silicon grating. Our design could achieve a sensitivity of more than 2.5%/m*g* with a tolerance of 5 nm and 2%/nm with a tolerance of 10 nm. The silicon grating and the Ag film should be patterned by electron beam lithography (EBL), whose linewidth should be smaller than 10 nm [33]. Hence, the acceleration measurement sensitivity can be as high as 2%/m*g* considering the tolerance of the period of silicon grating.

Figure 15b presents the acceleration sensitivity versus the thickness of the silicon grating with a variation of over 40 nm. The thickness of the silicon grating is determined by the thickness of the device layer of a SOI wafer, whose error could be controlled within 20 nm. Even with a tolerance of 20 nm, the maximal acceleration measurement sensitivity was greater than 2%/m*g*.

Similarly, Figure 15c,d demonstrate that our design was able to achieve sensitivity of more than 2%/m*g* with a tolerance of 20 nm for the thickness of the Ag film, and a tolerance of 0.01 for the duty cycle, which corresponds to a 7.55 nm variation for the period of the silicon grating. A 20 nm thickness control was achieved via evaporation, and the duty cycle tolerance was equivalent to the period tolerance. Therefore, acceleration sensitivity of 2%/m*g* could be obtained in practice by considering these tolerances (10 nm for EBL, 20 nm for device layer, and sacrificial layer thickness control). It is denoted by acceleration sensitivity (with tolerance) in Table 4.

Table 4 includes the final performance of the subwavelength grating pair-based nano-optomechanical accelerometer, which demonstrates that our scheme is a compelling design that holds promise for high performance and integration level. 

## 5. Conclusions

A scheme design for a high-performance nano-optomechanical accelerometer that can be fabricated by a single SOI chip was proposed in this paper. The accelerometer contains a subwavelength grating pair-based optical displacement readout and a rotated serpentine spring-mass micromachined structure. The micromachined structure transforms the acceleration to the displacement of the silicon grating and the optical readout measures the displacement with high sensitivity. The parameters of the optical readout, including the period and thickness of silicon grating, duty cycle, thickness of film, air gap, and wavelength, were optimized by combining a genetic algorithm and RCWA simulation. The optimized solution was demonstrated to have the high displacement sensitivity of 1.8%/nm through two methods. The mechanical structure was designed in terms of fabrication feasibility and target performance. The final optimal design holds promise for achieving extremely high acceleration measurement sensitivity of 2.8%/m*g*. As tolerance was considered during fabrication, sensitivity was still as high as 2%/m*g*, which outperforms previously reported counterparts. This design proved itself as a promising candidate as a high-performance optical inertial sensor, and the method and theory used in this work allow for the design of novel optomechanical devices.

## Figures and Tables

**Table 1:** Optimization interval and optimal values of the optical readout parameters.

Subwavelength Grating Parameters	Lower Limit	Upper Limit	Optimal Value
*p*	0.2 μm	2 μm	0.756 μm
*dc*	0.1	1	0.354
*λ*	500 nm	1550 nm	641 nm
*t*_1_	0.5 μm	2 μm	0.664 μm
*t_m_*	50 nm	200 nm	0.194 μm
*a_g_*	0.2 μm	2 μm	1.170 μm

**Table 2:** Dimensional parameters and spring constants of three types of springs with the same spring constant along the *x*-axis.

Dimensional Parameters	Value	Spring Constant	Value
*l*_1_	10 mm	$k_{x},\;k_{x}^{c},\;k_{x}^{str}$	~5.6 N/m
$l_{1}^{c}$	123.4 mm	$k_{t}^{r}$	3.73 × 10^−5^ Nm/°
$l_{2}^{c}$	0.4 mm	$k_{t}^{c}$	5.71 × 10^−6^ Nm/°
*l^str^*	22.9 mm	$k_{t}^{str}$	2.44 × 10^−5^ Nm/°

**Table 3:** Dimensional and performance parameters of the mechanical design.

Dimensional Parameter	Value
Bulky plate	5.2 mm × 5.2 mm × 0.664 μm
*l*_1_	25 μm
*l*_2_	28 μm
*l*_3_	4 μm
*l*_4_	4 μm
*w*	0.4 μm
*t*	0.664 μm
Acceleration-displacement sensitivity	1.56 nm/m*g*
Maximum stress (per *g*)	99.8 MPa
Resonant frequency	399.5 Hz

**Table 4:** Performance of our scheme design.

Performance	Value
Acceleration sensitivity (ideal)	2.8%/m*g*
Acceleration sensitivity (with tolerance)	2%/m*g*
Optical displacement sensitivity	1.8%/nm
Acceleration-displacement sensitivity	1.56 nm/m*g*
Maximum load (along the sensitive axis)	~ 1.5 *g* (10% of fracture limit) 16 *g* (100% of fracture limit)
Resonant frequency	~ 400 Hz

## Supplementary Materials

Supplementary Materials are available online at http://www.mdpi.com/1424-8220/18/7/2036/s1.
