# Supplementary material for: Single Chip-Based Nano-Optomechanical Accelerometer Based on Subwavelength Grating Pair and Rotated Serpentine Springs
Source: Sensors (Basel). 2018 Jun 26;18(7):2036. doi: 10.3390/s18072036 (PMC6068687; doi:10.3390/s18072036)
Supplement: Supplementary file 1 [file sensors-18-02036-s001.zip › sensors-304867-supplementary.pdf]

Scheme design for a single chip-based singel-axis nano-optomechanical accelerometer based on subwavelength grating pair and rotated serpentine springs

Qianbo Lu, Jian Bai, Kaiwei Wang, Peiwen Chen, Weidong Fang, Chen Wang and Guoguang Yang

State Key Laboratory of Modern Optical Instrumentation, Zhejiang University, Hangzhou 310027, China

I. Anisotropy of moncrystal silicon

The stiffness and compliance coefficient depend on crystal axis orientation in terms of the anisotropic Young's modulus, the shear modulus and Poisson's ratio. Because silicon has cubic diamond crystal structure, the lattice system is composed of three axes which can be described by three lattice vectors orthogonal and of equal length. The crystal axis coordinate system for crystal plane (100), which is commonly the device orientation of the device layer, is defined by the normal vector $\boldsymbol{e}_1$ = [100] and two orthogonal vectors $\boldsymbol{e}_2$ = [010] and $\boldsymbol{e}_3$ = [001], as illustrated in Figure S1(a). The stiffness coefficient matrix has the following structure with three independent elastic coefficients,

$$C_{100} = \begin{pmatrix} c_{11} & c_{12} & c_{12} & & & \\ c_{12} & c_{11} & c_{12} & & & \\ c_{12} & c_{12} & c_{11} & & & \\ & & & c_{44} & & \\ & & & & c_{44} & \\ & & & & & c_{44} \end{pmatrix}, \tag{S1}$$

where c_{11}, c_{12}, c_{44} are 165.7, 63.9 and 79.6 GPa, respectively [1]. Actually, some other literatures proposed slightly different values, but the difference is not significant and can be neglected [2]. Herein, we use the values proposed by Mason (1958) to make the computation. In ANSYS software, ones can input this anisotropic elastic matrix of stiffness form to get the final output with consideration of the anisotropy.

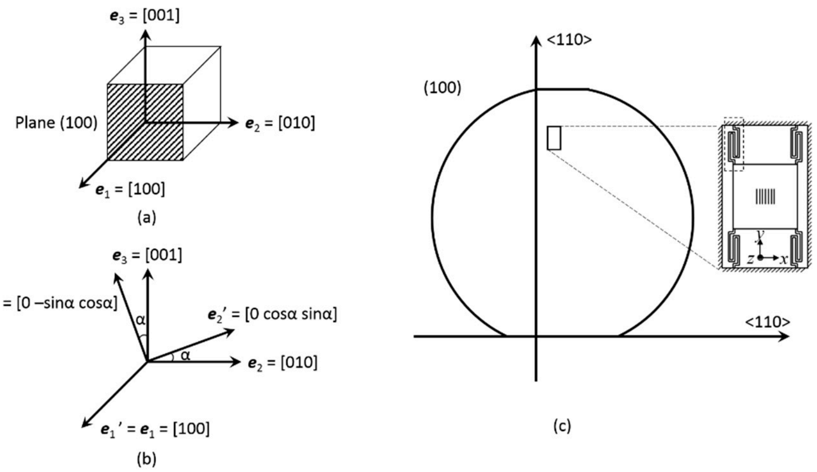

Figure S1. (**a**) Crystal-axis coordinate system for crystal plane (100); (**b**) new coordinate system for crystal plane (100) with a rotation of α. (c) crystal orientation of the SOI wafer.

For the analytical calculation, we should get the expressions of Young's modulus, shear modulus and Poisson's ratio first. We thus obtain the compliance matrix which is the inverse of the stiffness matrix:

$$S_{100} = \begin{pmatrix} s_{11} & s_{12} & s_{12} & & & \\ s_{12} & s_{11} & s_{12} & & & \\ s_{12} & s_{12} & s_{11} & & & \\ & & & s_{44} & & \\ & & & & s_{44} & \\ & & & & & s_{44} \end{pmatrix}, \quad \text{(S2)}$$

In which $s_{11} = (c_{11} + c_{12})/[(c_{11} - c_{12})(c_{11} + 2c_{12})] = 7.68$, $s_{12} = -c_{12}/[(c_{11} - c_{12})(c_{11} + 2c_{12})] = -2.14$, $s_{44} = 1/c_{44} = 12.56 \times 10^{-12}$ Pa^{-1}. According to Nye, Wortman and Brantley's work [2-3], we can finally get the expressions of Young's modulus, shear modulus and Poisson's ratio in any crystallographic direction:

$$\begin{aligned} E_{hkl} &= 1/\left(s_{11} + \left(s_{11} - s_{12} - 0.5s_{44}\right)\left(m^4 + n^4 + p^4 - 1\right)\right), \\ G_{ij} &= 1/\left(s_{44} + 4\left(s_{11} - s_{12} - 0.5s_{44}\right)\left(m_i^2 m_j^2 + n_i^2 n_j^2 + p_i^2 p_j^2\right)\right) \quad i \neq j, \\ v_{ij} &= -\frac{s_{12} + \left(s_{11} - s_{12} - 0.5s_{44}\right)\left(m_i^2 m_j^2 + n_i^2 n_j^2 + p_i^2 p_j^2\right)}{s_{11} - 2\left(s_{11} - s_{12} - 0.5s_{44}\right)\left(m_i^2 n_i^2 + n_i^2 p_i^2 + p_i^2 m_i^2\right)} \quad i \neq j, \end{aligned} \quad \text{(S3)}$$

Where $[h\ k\ l]$ is the normal vector of surface $(h\ k\ l)$, m, n, p are the direction cosines for the calculated direction, which can be described as:

$$\begin{aligned} m &= h/\left(h^2 + k^2 + l^2\right)^{0.5}, \\ n &= k/\left(h^2 + k^2 + l^2\right)^{0.5}, \\ p &= l/\left(h^2 + k^2 + l^2\right)^{0.5}. \end{aligned} \quad \text{(S4)}$$

i and *j* denote the crystal axes, as per Figure S1, G_{ij} represents the ratio of shear stress to the shear strain involving axis $\boldsymbol{e}'_i$ and $\boldsymbol{e}'_j$, v_{ij} is the Poisson's ratio which represents the strain variation in the direction $\boldsymbol{e}'_j$ when there is a strain variation applied in the direction $\boldsymbol{e}'_i$, (m_i, n_i, p_i) and (m_j, n_j, p_j) are the direction cosines for $\boldsymbol{e}'_i$ and $\boldsymbol{e}'_j$ directions. According to Equation S3 and Equation S4, we can obtain the anisotropic mechanical properties for (100) plane. We plot the Young's modulus ($E_{//}$, $E_{\perp}$), shear modulus ($G_{//}$, $G_{\perp}$) as well as Poisson's ratio ($v_{//}$, $v_{\perp}$) both normal and parallel to the crystal surface (100) versus the angle α shown in Figure S1(b).

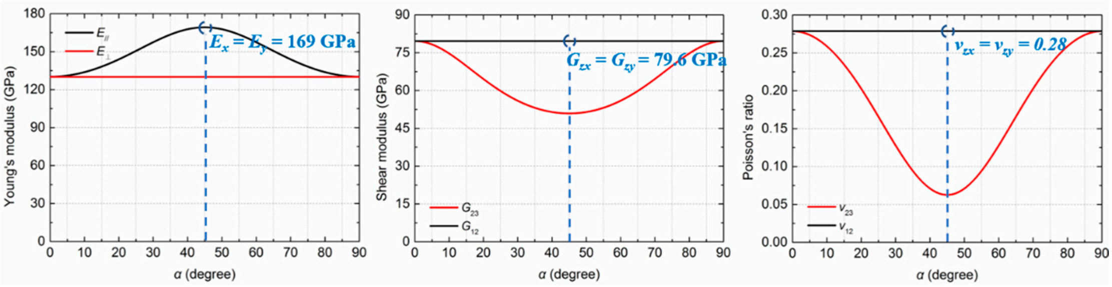

(**a**) (**b**) (**c**)

Figure S2. (**a**) Young's modulus in the direction $\boldsymbol{e_1}'$ and $\boldsymbol{e_2}'$; (**b**) shear modulus and (**c**) Poisson's ratio in the direction *zx* and *xy* for (100) silicon. The coordinate system is defined in Figure S1(b).

When it comes to our design proposed in the manuscript, the *x*-axis and *y*-axis are parallel to the <110> direction; *z*-axis is parallel to the <100> direction, as shown in Figure S1(c). Hence, the anisotropic mechanical properties can be given as below: $E_x = E_y = 169$ GPa, $E_z = 130$ GPa; $G_{zx} = G_{zy} = 79.6$ GPa, $G_{xy} = G_{yx} = 50.9$ GPa; $v_{zx} = v_{zy} = 0.28$, $v_{zx} = v_{zy} = 0.06$. Based on the spring constant derivation, we can easily find that the spring constant expression only involves the Young's modulus along the *x*-axis (or *y*-axis). Hence, $E_x = 169$ GPa is used in the calculation of the spring constant in the manuscript. For the torsion constant, it is related to G_{zx}, which is used in the manuscript.

II. Spring constant derivation

The energy method indicates that if a deformable structure in equilibrium under the action of a system of loads is given a small virtual deformation, then the virtual work done by the external loads equals the virtual work done by the internal forces [4]. The spring constant is defined as $k_{ij} = F_j/\Delta_i$ or $k_{\theta i\theta j} = M_j/\theta_i$ and can be arranged in a 6 × 6 matrix. Under the assumption that there are no variations along the *z*-axis, no coupling between in-plane and out-of-plane direction for the thin beam [5], the number of nonzero terms reduces to 12 since $k_{ij} = k_{ji}$. In the matrix on the right-hand side, the second subscript is dropped when it is identical to the first one.

$$k = \begin{pmatrix} k_{xx} & k_{xy} & k_{xz} & k_{x\theta_x} & k_{x\theta_y} & k_{x\theta_z} \\ k_{yx} & k_{yy} & k_{yz} & k_{y\theta_x} & k_{y\theta_y} & k_{y\theta_z} \\ k_{zx} & k_{zy} & k_{zz} & k_{z\theta_x} & k_{z\theta_y} & k_{z\theta_z} \\ k_{\theta_x x} & k_{\theta_x y} & k_{\theta_x z} & k_{\theta_x\theta_x} & k_{\theta_x\theta_y} & k_{\theta_x\theta_z} \\ k_{\theta_y x} & k_{\theta_y y} & k_{\theta_y z} & k_{\theta_y\theta_x} & k_{\theta_y\theta_y} & k_{\theta_y\theta_z} \\ k_{\theta_z x} & k_{\theta_z y} & k_{\theta_z z} & k_{\theta_z\theta_x} & k_{\theta_z\theta_y} & k_{\theta_z\theta_z} \end{pmatrix} \Rightarrow \begin{pmatrix} k_x & k_{xy} & & & & k_{x\theta_z} \\ k_{yx} & k_y & & & & k_{y\theta_z} \\ & & k_z & k_{z\theta_x} & k_{z\theta_y} & \\ & & k_{\theta_x z} & k_{\theta_x} & k_{\theta_x\theta_y} & \\ & & k_{\theta_y z} & k_{\theta_y\theta_x} & k_{\theta_y} & \\ k_{\theta_z x} & k_{\theta_z y} & & & & k_{\theta_z} \end{pmatrix}. \tag{S5}$$

For the spring constant of a rotated serpentine spring with a guided end, what we concern most is the spring constant along the *x*-axis for the rotated serpentine spring, $k_x^r = F_x/d_x$. From the constraints that the sum of the rotation around the *z*-axis caused by the F_x load and an unknown reaction M_{rz} (here is moment) is zero, we can write:

$$\theta_{z1} + \theta_{z2} = \frac{F_x}{k_{\theta_z x}} + \frac{M_{rz}}{k_{\theta_z}} = 0. \tag{S6}$$

Hence, the moment of the reaction M_{rz} can be expressed as

$$M_{rz} = -\frac{F_x k_{\theta_z}}{k_{\theta_z x}}. \tag{S7}$$

The displacement d_x due to the load F_y and moment M_{rz} applied to the free end of the spring can be calculated by substituting M_{rz}

$$d_x = \frac{F_x}{k_x} + \frac{M_{rz}}{k_{x\theta_z}} = \frac{k_{x\theta_z} k_{\theta_z x} - k_x k_{\theta_z}}{k_x k_{x\theta_z} k_{\theta_z x}} F_x. \tag{S8}$$

Therefore, the spring constant of the rotated serpentine spring with a guided end along the x-axis can be written as

$$k_x{}^r = \frac{F_x}{d_x} = \frac{k_{x\theta_z}{}^2 k_x}{k_{x\theta_z}{}^2 - k_{\theta_z} k_x}. \tag{S9}$$

The expressions for the spring constant elements including k_x, $k_{\theta z}$ and $k_{x\theta z}$ can be obtained by the unit-load method, as per Figure S3.

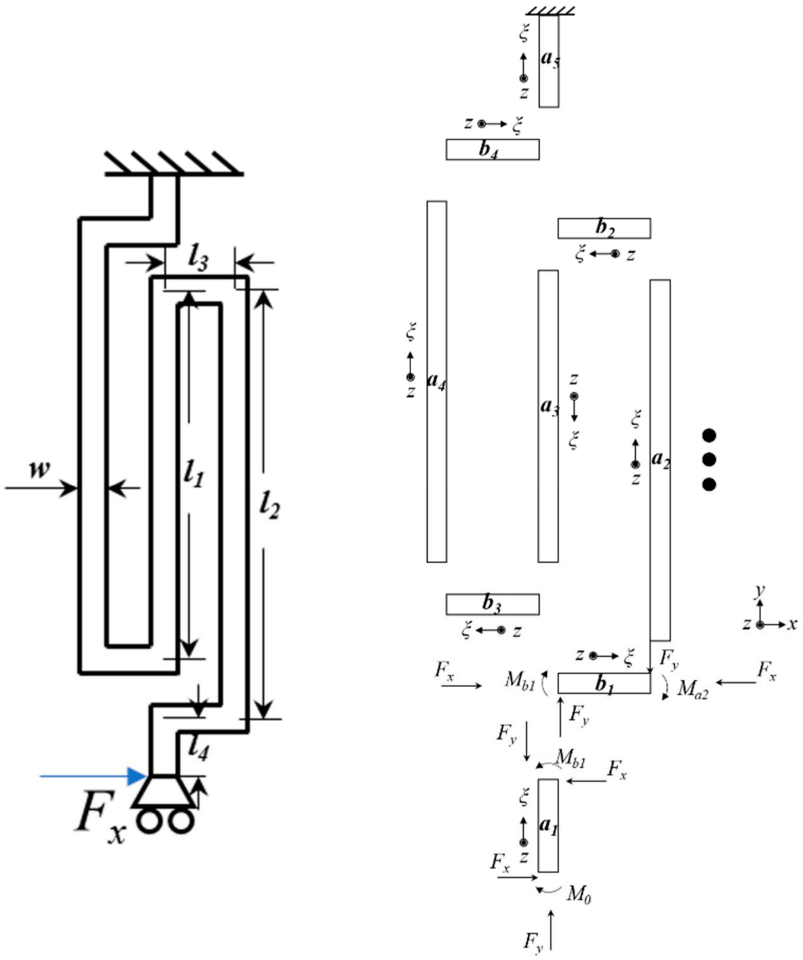

Figure S3. Free-body diagram of a rotated serpentine spring with a guided-end.

$$\theta_z = 2\int_0^{l_4} \frac{M_z}{EI_z} dz + 4\int_0^{l_3} \frac{M_z}{EI_z} dz + 2\int_0^{l_2} \frac{M_z}{EI_z} dz + \int_0^{l_1} \frac{M_z}{EI_z} dz$$
$$\Rightarrow \theta_z = M_z \frac{2l_4 + 4l_3 + 2l_2 + l_1}{EI_z}. \quad \text{(S10)}$$

In which I_z is the moment of inertia along the z-axis, which is $w^3t/12$. Thus, we can derive the $k_{\theta z}$

$$k_{\theta_z} = M_z / \theta_z = EI_z / \left(2l_4 + 4l_3 + 2l_2 + l_1\right). \quad \text{(S11)}$$

For k_x, we can write the moment of each segment

$$
\begin{aligned}
M_{a1} &= M_0 - F_x\xi, \\
M_{b1} &= M_0 - F_x l_4 + F_y\xi, \\
M_{a2} &= M_0 - F_x l_4 + F_y l_3 - F_x\xi, \\
M_{b2} &= M_0 - F_x\left(l_4 + l_2\right) + F_y l_3 - F_y\xi, \\
M_{a3} &= M_0 - F_x\left(l_4 + l_2\right) + F_x\xi, \\
M_{b3} &= M_0 - F_x\left(l_4 + l_2 - l_1\right) - F_y\xi, \\
M_{a4} &= M_0 - F_x\left(l_4 + l_2 - l_1\right) - F_y l_3 - F_x\xi, \\
M_{b4} &= M_0 - F_x\left(l_4 + 2l_2 - l_1\right) - F_y l_3 + F_y\xi, \\
M_{a5} &= M_0 - F_x\left(l_4 + 2l_2 - l_1\right) - F_x\xi,
\end{aligned}
\tag{S12}
$$

The total energy in the rotated serpentine spring is

$$
\begin{aligned}
U = &\int_0^{l_4}\frac{M_{a1}{}^2}{2EI_z}d\xi + \int_0^{l_3}\frac{M_{b1}{}^2}{2EI_z}d\xi + \int_0^{l_2}\frac{M_{a2}{}^2}{2EI_z}d\xi + \int_0^{l_3}\frac{M_{b2}{}^2}{2EI_z}d\xi \\
&+ \int_0^{l_1}\frac{M_{a3}{}^2}{2EI_z}d\xi + \int_0^{l_3}\frac{M_{b3}{}^2}{2EI_z}d\xi + \int_0^{l_2}\frac{M_{a4}{}^2}{2EI_z}d\xi + \int_0^{l_3}\frac{M_{b4}{}^2}{2EI_z}d\xi + \int_0^{l_4}\frac{M_{a5}{}^2}{2EI_z}d\xi.
\end{aligned}
\tag{S13}
$$

By applying the displacement (along the *y*-axis) and rotation boundary conditions we can obtain

$$
\begin{aligned}
\Delta_y &= \frac{\partial U}{\partial F_y} = 0 \\
\theta_0 &= \frac{\partial U}{\partial M_0} = 0
\end{aligned}
\tag{S14}
$$

Bring the partial derivation inside the integrals of Equation S13, we can obtain k_x combined with the moment relations

$$
\begin{aligned}
k_x &= \frac{F_x}{\Delta_x} = \frac{F_x\partial F_x}{\partial U} \\
&= 3EI_z \Big/ \left(\begin{array}{l} 3l_3\left(l_4{}^2 + \left(l_4 + l_2 - l_1\right)^2 + \left(l_4 + l_2\right)^2 + \left(l_4 + 2l_2 - l_1\right)^2\right) \\ +2\left(\left(l_4 + l_2\right)^3 - \left(l_4 + l_2 - l_1\right)^3\right) + \left(2l_4 + 2l_2 - l_1\right)^3 \end{array}\right).
\end{aligned}
\tag{S15}
$$

Similarly, using boundary conditions of $\Delta_y = 0, \Delta_x = 0$, we can obtain

$$
\begin{aligned}
k_{\theta_z x} &= \frac{F_x}{\theta_z} \\
&= 2EI_z / \left(2l_1\left(2l_4 + 2l_2 - l_1\right) + \left(2l_4 + 2l_2 - l_1\right)^2 + 2l_3\left(4l_4 + 4l_2 - 2l_1\right)\right).
\end{aligned}
\tag{S16}
$$

Substituting Equation S11, S15, and S16 into Equation S9, we can finally get the analytical expression of the spring constant along the *x*-axis for the rotated serpentine spring. The method we used is energy method and the unit-load method, which can be easily applied in analysis

of micromechanical flexures with different geometries. The flow-chart for spring constant analysis using energy methods is provided below.

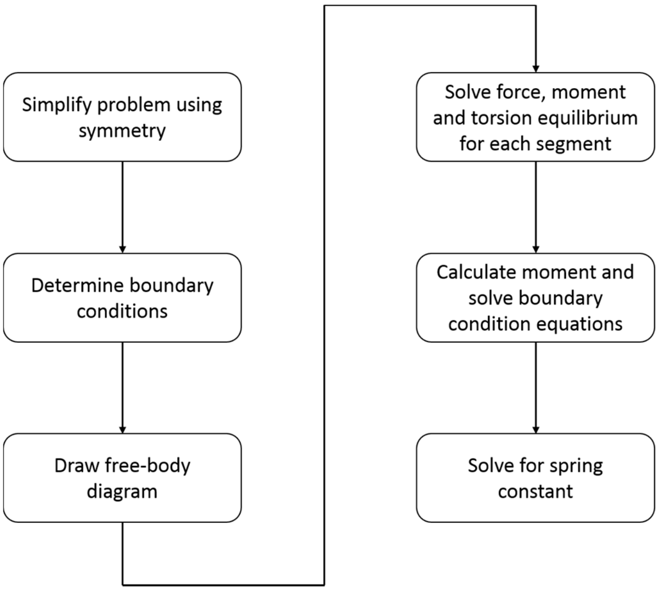

Figure S4. Flow-chart for spring constant calculation using energy method.

III. Torsion constant of two types of serpentine springs

For the classical serpentine spring, we can draw the free-body diagram, as shown in Figure S5. By using the unit-load method, we have

$$\theta_y = \int_0^{l_3^c} \frac{M_y}{GJ} dy + \int_0^{l_1^c/2} \frac{M_y}{EI_x} dy + \int_0^{l_2^c} \frac{M_y}{GJ} dy + \int_0^{l_1^c} \frac{M_y}{EI_x} dy + \int_0^{l_2^c} \frac{M_y}{GJ} dy + \int_0^{l_1^c/2} \frac{M_y}{EI_x} dy + \int_0^{l_3^c} \frac{M_y}{GJ} dy, \tag{S17}$$

where $I_x = wt^3/12$, $J \approx 2.25(w/2)^4$ because $w = t$. Simplifying Equation S17, the following equation is obtained

$$\theta_y = M_y \left[\frac{2l_1^c}{EI_x} + \frac{2\left(l_2^c + l_3^c\right)}{GJ} \right]. \tag{S18}$$

Hence, we can finally obtain the torsion constant of the classical serpentine spring

$$k_t^c = \frac{M_y}{\theta_y} = \left[\frac{2l_1^c}{EI_x} + \frac{2\left(l_2^c + l_3^c\right)}{GJ} \right]^{-1}. \tag{S19}$$

Because l_1^c is far larger than l_2^c and l_3^c, the second term of Equation S19 can be dropped, so it is simplified to

$$k_t^c \approx \frac{EI_x}{2l_1^c} = \frac{Ewt^3}{24l_1^c}. \tag{S20}$$

Similarly, we can get the torsion constant of the rotated serpentine spring from Figure S3

$$k_t^c = \left[\frac{4l_3}{EI_x} + \frac{3l_1 + 2(l_2 - l_1) + 2l_4}{GJ} \right]^{-1}. \tag{S21}$$

Under the premise that l_1 *is much larger than* $l_2 - l_1$, l_3 *and* l_4, the first term in Equation S21 can be dropped; thus, we get

$$k_t^c \approx \frac{GJ}{3l_1} = \frac{3Gw^3t}{64l_1}. \tag{S22}$$

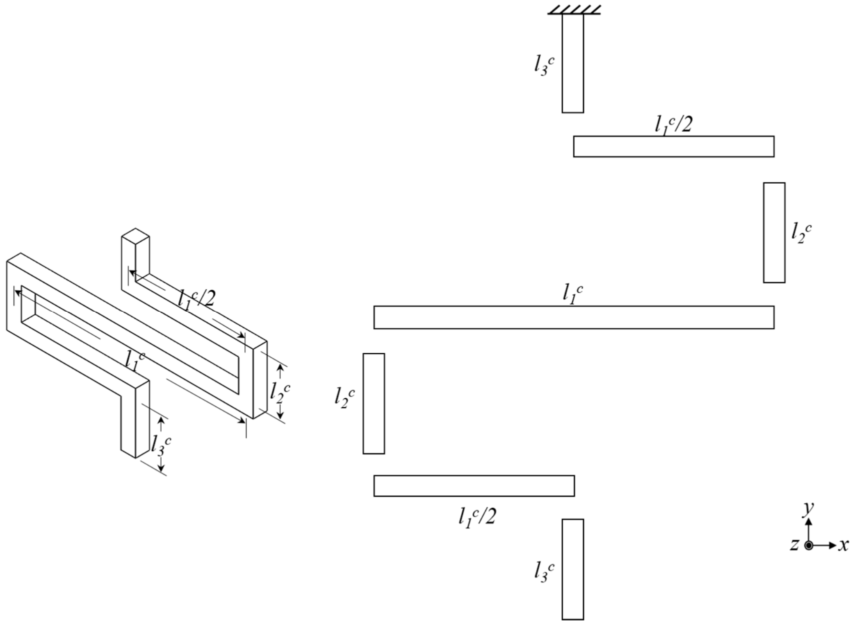

Figure S5. Free-body diagram of a classical serpentine spring with a guided-end.

IV. Feasible process flow

a) is the starting mirror polished SOI wafer; b) spin coat the electron beam resist; c) make the pattern of grating lines by electron beam lithography (EBL) and etch the device layer to the buried oxide layer; d) release the grating area by buffered oxide etch (BOE); e) deposit Ag and then use lift-off to pattern the upper metal film; f) also use EBL and ICP to pattern and etch the springs; g) release the suspended springs by BOE; h) release the proof mass to make the whole device suspended.

Notably, the release of proof mass should be placed after the release of the springs so that the release-related anti-stiction condition can hold. In order to release the bulky proof mass, some through-silicon vias can be introduced in advance.

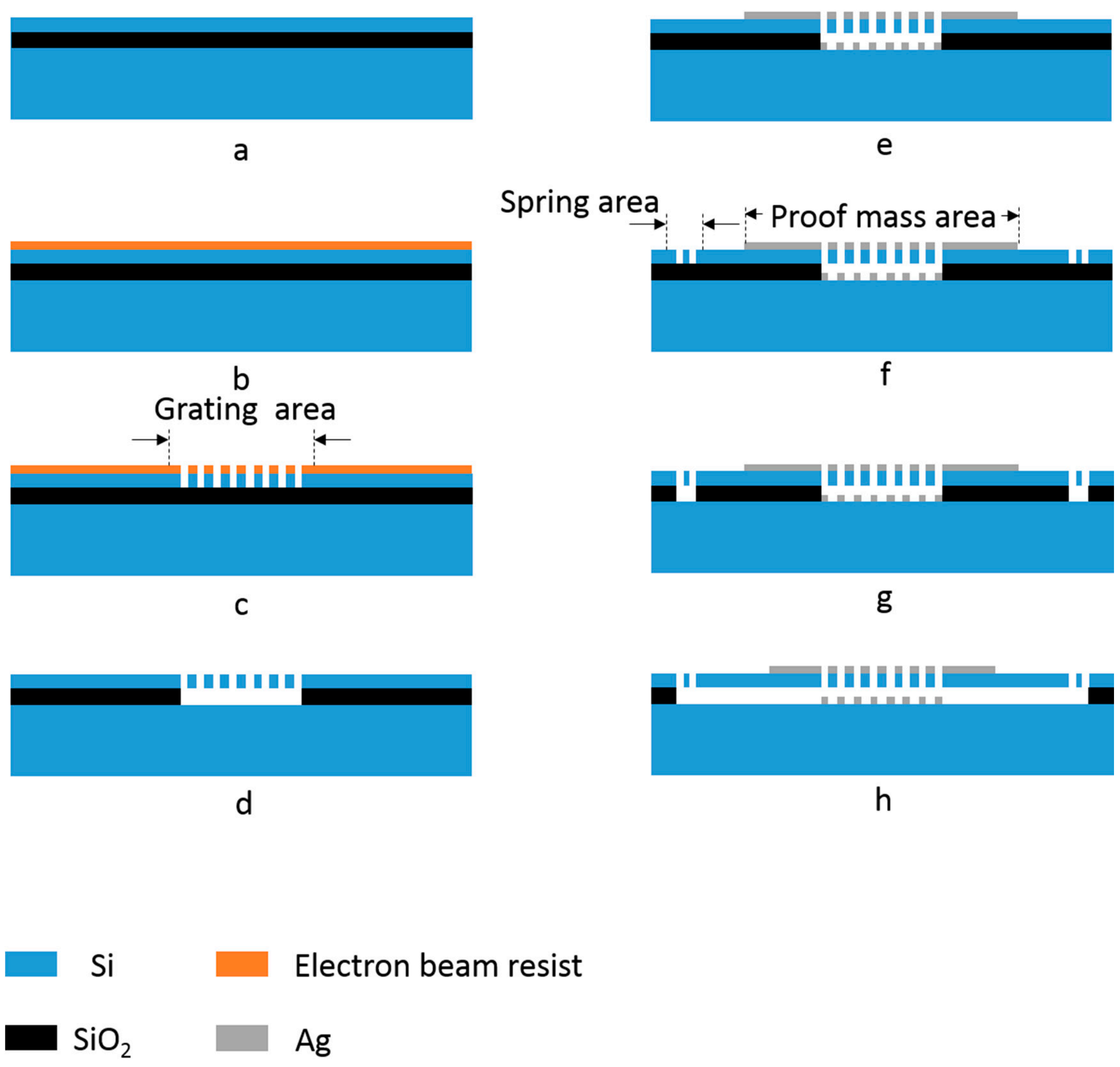

Figure S6. Process flow for fabrication the micromachined structure.

1. Mason, W. P., *Physical Acoustics and the Properties of Solids*. Princeton: Van Nostrand, 1958.
2. Wortman, J. J.; Evans, R. A., Young's modulus, shear modulus, and Poisson's ratio in silicon and germanium **1965**. *Journal of Applied Physics*, 36, (1), 153-156.
3. Nye, J. F., *Physical Properties of Crystals: Their Representation by Tensors and Matrices*. Oxford University Press, 1957.
4. Gere, J. M., Timoshenko, S. P., *Mechanics of Materials* 3rd end. London: Chapman and Hall, 1991.
5. Iyer, S.; Zhou, Y.; Mukherjee, T., Analytical modeling of cross-axis coupling in micromechanical springs. *Technical Proc. 1999 Int. Conf. on Modeling and Simulation of Microsystems*, 1999; 632–635.
